# NEFL is associated with inhibition of odontoblastic process in odontohypophosphatasia

**DOI:** 10.1007/s00774-026-01703-5

**Published:** 2026-03-14

**Authors:** Akira Nozoe, Yasuhisa Ohata, Makoto Fujiwara, Kenichi Yamamoto, Toshihiko Nambara, Chiho Nakano, Kazuaki Miyagawa, Mikihiko Kogo, Takeshi Taketani, Takuo Kubota, Yasuji Kitabatake, Susumu Tanaka, Keiichi Ozono

**Affiliations:** 1 Department of Oral and Maxillofacial Surgery, Graduate School of Dentistry, The University of Osaka, Osaka, Japan; 2 Department of Pediatrics, Graduate School of Medicine, The University of Osaka, Osaka, Japan; 3 Laboratory of Children’s health and Genetics, Division of Health Science, Osaka University Graduate School of Medicine, Osaka, Japan; 4 Department of Pediatrics, Faculty of Medicine, Shimane University, Shimane, Japan; 5 Center for Promoting Treatment of Intractable Diseases, ISEIKAI International General Hospital, Osaka, Japan

**Keywords:** Odontohypophosphatasia, Odontoblast, Osteoblast, Induced pluripotent stem (iPS) cells

## Abstract

**Introduction:**

Hypophosphatasia (HPP) is a rare bone disease caused by pathological variants of *ALPL*. While hypomineralization of dentin and odontoblast (OD) differentiation defects are known to occur in HPP, the underlying pathophysiology remains poorly understood.

**Materials and methods:**

We generated induced pluripotent stem cell (iPSC) lines from patients with perinatal severe HPP (Perinatal) and then isogenic (Rescued) and odontohypophosphatasia type (Odonto) lines using gene editing. These three HPP-iPSC lines were differentiated into OD-like cells via mesenchymal stem cells derived from neural crest cells. The characteristics of the OD-like cells were assessed.

**Results:**

Rescued-OD-like cells demonstrated mineralization ability, increased *microtubule-associated protein tau* (*MAPT*) expression, and unidirectional cell processes, while these characteristics were impaired in Perinatal- and Odonto-OD-like cells. These findings suggest that these features are associated with reduced ALP activity. Notably, *neurofilament light chain* (*NEFL*) expression was enhanced in Odonto-OD-like cells compared to Perinatal- and Rescued-OD-like cells. *NEFL* knockdown partially rescued cell process elongation in Odonto-OD-like cells without affecting alkaline phosphatase activity, while *NEFL* overexpression inhibited cell process elongation in Rescued-OD-like cells.

**Conclusions:**

Enhanced expression of *NEFL* in Odonto-OD-like cells negatively correlates with OD morphology and may be relevant to odontoblast morphological abnormalities associated with odontohypophosphatasia; however, generalization to other odonto-HPP genotypes requires additional patient-derived lines.

**Supplementary Information:**

The online version contains supplementary material available at 10.1007/s00774-026-01703-5.

## Introduction

Hypophosphatasia (HPP), a rare bone mineral disease, is caused by pathogenic variants in the *ALPL* gene that reduce alkaline phosphatase (ALP) activity [1]. Perinatal severe HPP (Perinatal), the type of the most reduced ALP activity, is characterized symptoms such as severe hypomineralization of the skeleton and teeth development in fetuses and neonates. In contrast, odontohypophosphatasia (Odonto), mildly reduced ALP activity, is characterized by dental problems without skeletal manifestations [2]. Almost all patients with HPP exhibit dental manifestations. The dental findings in HPP are as follows: early loss of deciduous teeth without root resorption due to cementum defects before the age of four, thinning of the dentin, shortening of tooth roots, and wide pulp chambers in the oral cavity [3]. Perinatal can disturb the dentoalveolar complex more broadly; nationwide Japanese surveys reported that severe/non‑Odonto phenotypes including Perinatal cases frequently show tooth hard‑tissue hypomineralization and malocclusion/oral functional problems, whereas tooth/enamel hypomineralization is uncommon in Odonto cases [4, 5]. However, because hypomineralization of dentin and enamel has been reported in Odonto, it is important to keep potential dental problems in mind in patients with Odonto [6]. In addition, it is likely that hypomineralization of the mantle dentin causes poor attachment of the cementum to the dentin [7]. Several studies have reported inhibition of odontoblast (OD) differentiation and hypomineralization of mantle dentin in patients with HPP [8, 9]. Dental tissue specimens revealed reduced root dentin in a model mouse of Odonto [10]. Odontoblastic processes were lost in *Alpl*-knockout mice [11]. Therefore, elucidating the pathophysiology of abnormal dentin in patients with HPP will help in the development of novel therapies for teeth with HPP.

Odontoblasts play a pivotal role in dentin formation and are derived from dental mesenchymal stem cells (MSC) which originate from cranial neural crest cells (NCC) [12]. Dentin forms the bulk of teeth, and the root dentin is covered with cementum which is implicated in the attachment of teeth to the alveolar bone [13]. Although there are no established differentiation markers for ODs, increased expression of dentin sialophosphoprotein (DSPP) and nestin (NES) are features of ODs [14]. In addition, ODs express the microtubule-associated protein tau (MAPT), a known cell process formation marker [14]. Furthermore, ODs show elongation of the odontoblastic process in one direction, which is not observed in osteoblasts (OBs) [15–17]. Neurons are well known for the elongation of cell process, and neurofilament light chain (NEFL) has been reported to be involved in the cell process formation of neurons [18]. NEFL is also expressed in dental pulp cells [19]. The elongation of cell processes is essential in ODs for the formation and mineralization of dentin [20]. Although a method for isolating ODs from human teeth has been reported, it remains challenging to maintain the extracted cells with a high degree of purity [21]. Previous studies have reported successful direct induction of OD-like cells from human embryonic stem (ES) cells and induced pluripotent stem cells (iPSC) [22, 23]. However, there is no report about OD-like cells differentiation from human iPSC via NCC which recapitulates the physiological differentiation of ODs. Recently, the investigation of disease-specific iPSC has the potential to advance our understanding of the pathology of rare disorders, facilitate targeted drug discovery, and avoid ethical problems specific to ES cell research [24]. The effect of the genetic background for the phenotype can be avoided by making the pathogenic variant-rescued iPSC from each iPSC line as an isogenic control [25]. Multiple types of cells from the same iPSC line of the same patient can be analyzed if the appropriate induction methods for each type of cell are available. Our previous study successfully elucidated the pathophysiology of OB-like cells differentiated from iPSCs in patients with HPP (HPP-iPSC) using gene editing [26].

In this study, to investigate mechanisms underlying OD differentiation defects, odontoblastic process abnormalities, and altered mineralization capacity in Perinatal and Odonto, we investigated isogenic HPP-iPSC lines, a gene-repaired line and an Odonto line, with the same genetic background using gene editing in iPSC derived from a patient with Perinatal, and we compared the OD-like cell differentiation from the three HPP-iPSC lines. This study suggested a specific pathophysiology of ODs in patients with Odonto and will provide an opportunity to develop tooth-specific treatments for patients with HPP.

## Materials and methods

### Generation of human iPSCs and differentiation of OD-like cells

The present study, including the experimental procedures for skin biopsy and iPSC production, was approved by the Osaka University Graduate School of Medicine and Shimane University Medical Hospital ethical committee [approval numbers: 13123 (823)-4 and 1110], and written informed consent was obtained from the parents or legal guardians of the patients in accordance with the Declaration of Helsinki.

Dermal fibroblasts from three healthy humans were isolated and used to establish iPSCs [26–28]. This study included a Japanese male patient with Perinatal who was homozygous for the c.1559delT pathogenic variant in *ALPL* gene. Human dermal fibroblasts from the patient with Perinatal were obtained when the patient was 6 months old, and they were used to establish iPSCs. Odontogenic differentiation from iPSCs was performed as previously reported [22]. Briefly, NCC were induced from iPSCs with a chemically defined medium containing 10 μM SB431542 (Selleck Chemicals) and 1 μM CHIR99021 (LC Laboratories), sorted using an anti-p75 antibody (BD Biosciences) with FACS, and differentiated into MSC in αMEM, 10% FBS, 5 ng/ml FGF2 (Wako), 25 U/ml penicillin, and 25 μg/ml streptomycin. MSC were differentiated into OD-like cells in DMEM/F-12 (Wako), 10% FBS, recombinant human BMP4 (R and D), recombinant human/mouse FGF-8b Protein (R and D), 0.1 mM dexamethasone (Wako), 35 mg/ml ascorbic acid (Wako), and 10 mM β-glycerophosphate (Sigma) on gelatin (Nacalai Tesque)-coated plates for 3 weeks. As a control, the same MSC were differentiated into OB-like cells in αMEM, 10% FBS, 0.1 mM dexamethasone, 50 mg/ml ascorbic acid, and 10 mM β-glycerophosphate on laminin-coated plates for 3 weeks. We identified MSC as day 0 for OD-like cell and OB-like cell differentiation.

### Gene editing for Rescue model using CRISPR-Cas9 system

Guide RNA (gRNA) for CRISPR-Cas9 was designed using the CRISPOR software. gRNAs were cloned into the px330 vector using the in-fusion method. To perform gene editing of perinatal severe HPP-iPSCs (Perinatal-iPSCs), colonies of the obtained Perinatal-iPSCs were dissociated into single cells using TrypLE Express (Life Technologies) with 10 μM ROCK inhibitor (Y27632; Reagents Direct). The cells were then mixed with CRISPR-Cas9 and the donor vector and electroporated using the Neon Transfection System (Life Technologies). The treated cells were plated onto 10-cm dishes. Puromycin (1 μg/ml) was added to the medium 4 days after transfection, and selected clones with the cassette were expanded after PCR check. For cassette deletion in iPSCs, 1 × 10⁶ iPSCs cultured with 0.5 μg/ml puromycin were transfected with 8 μg Cre expression vector. After transfection, cells were re-plated on the medium and incubated to harvest single colonies. After 5 days of transfection, 2 μM 1-[2-deoxy, 2-fluoro-8-d-arabinofuranosyl]-5-iodouracil was added to the medium, and the obtained colonies were selected using genomic DNA (gDNA) analysis to confirm that both allele-repaired iPS cells (Rescued-iPSCs) could be generated from perinatal-iPSCs.

### Gene editing for odontohypophosphatasia model using Prime editing

Prime editing gRNA (pegRNA) was designed to create an odontohypophosphatasia iPSC model (Odonto-iPSCs). Since c.550C>T (p.Arg184Trp) has been reported to exert a dominant-negative effect and to cause Odonto-HPP in heterozygous individuals, we selected the pathogenic variant of Odonto who was heterozygous for the c.550C>T (p. Arg184Trp) pathogenic variant in *ALPL* gene using reported reference data and a list of patients with Odonto who were followed up at the Osaka University Hospital (unpublished data) [2]. The pegRNA for prime editing was designed using the PrimeDesign software. The gRNAs were cloned into the pU6-pegRNA-GG-Vector using Golden Gate Assembly. Colonies of obtained Rescued-iPSCs were dissociated into single cells using TrypLE Express (Life Technologies) with 10 μM ROCK inhibitor (Y27632; Reagents Direct, Encinitas). Following this, 4.0 × 10^5^ cells were mixed with the synthetic pegRNA (7.2 μg) and PEmax (2 μg) and electroporated using the Neon Transfection System (settings: 1200 V, 20 ms, 2 pulse) (Life Technologies). Electroporated Rescued-iPSCs were plated onto 10-cm dishes. Blasticidin (10 ng/μl) was added to the medium a day after transfection, and clones were selected using gDNA analysis to confirm that Odonto-iPSCs could be generated from Rescued-iPSCs.

### Immunofluorescence staining

Mesenchymal stem cells (MSC), Odontoblast (OD)-like cells, and osteoblast (OB)-like cells were plated on a 4-well Slide and Chamber (WATSON 192-004) at a density of 5.0 × 10^3^ cells/well in triplicate and cultured in MSC maintenance medium and OD-like cells and OB-like cells induction medium. After 4 days of MSC, OD-like cells and OB-like cells incubation, each cell was washed with PBS thrice, fixed with 4% paraformaldehyde at room temperature for 10 min, and washed with PBS thrice. Next, the cells were incubated with 0.1% Triton-X buffer at room temperature for 10 min and washed three times with PBS. Cells were incubated with DSPP antibody diluted to 1:100 in blocking buffer (80% PBS and 20% Blocking one) at 4 ℃ overnight. After washing three times with PBS, phalloidin (Abcam) was diluted to 1:1000 in blocking buffer at room temperature for 1 h. After washing with PBS thrice, the cells were incubated with Hoechst 33342 solution (Dojindo) diluted to 1:100 in blocking buffer and goat anti-mouse IgG H and L (Alexa Fluor^®^ 594) antibody diluted 1:500 in blocking buffer at room temperature for 1 h. After washing with PBS thrice, all the slides were visualized and imaged using an FV3000 fluorescent microscope (Olympus).

### The measurement of cell processes

After immunofluorescence staining for HPP-OD-like cells, we measured the distance from the tips of the cell process to the nucleus in all cell in the microscopic field of view, excluding overlapping cells, using IMARIS software and a previously reported method [29].

### Reverse transcription quantitative polymerase chain reaction assay

Total RNA was extracted using the RNeasy Mini Kit, and cDNA was synthesized from total RNA using the ReverTra Ace qPCR RT Master Mix, according to manufacturer’s instructions. Reverse transcription quantitative polymerase chain reaction (RT-qPCR) was performed with a set of specific primers using THUNDERBIRD SYBR qPCR Mix and the QuantStudio 7 Flex Real-time PCR System. Quantification of gene expression was based on the 2^−ΔΔ^Ct method and normalized to *ACTB* gene expression.

### ALP assay

ALP activity was measured using the LabAssay^™^ ALP kit, according to manufacturer’s instructions. ALP activity was determined as nmol/h phosphorylated nitrophenol release in the presence of ALP and further normalized to the amount of protein.

### Western blotting

MSC were differentiated into OD-like cells and OB-like cells for 3 weeks. Total protein was extracted from the induced cells using RIPA buffer containing a protease and inhibitor cocktail. After centrifugation at 15,000 *g* and 4 °C for 15 min, supernatants were stored at −80 °C. The protein concentration was measured using a DC assay reagent. Proteins were subjected to SDS-PAGE and transferred to an immunoblotting polyvinylidene fluoride membrane. After blocking, the membrane was incubated with primary antibody against DSPP (1:100), NES (1:1000), OB-cadherin (1:1000), and HRP-conjugated anti-β-actin antibody (1:5000). After incubation with the secondary antibody, the signals were visualized using a chemiluminescence system. Densitometry was quantified using Imagelab software.

### Alizarin red staining

Mineralization of the cell layers was assessed by Alizarin Red staining. The cell layer was washed with PBS, fixed with 10% neutral-buffered formalin at room temperature for 10 min, washed with water, stained with 2% Alizarin Red S at room temperature for 30 min, and washed several times with water. The calcium content of the cell layers was quantified using a calcium assay kit, according to manufacturer’s instructions. Briefly, each cell layer was washed with PBS and scraped off. Calcium in the cell layer was extracted with 0.1 M HCl at room temperature for 1 h on a rotary shaker, centrifuged at 10,000 *g* for 10 min, and the supernatant was used as the sample after neutralization with NaOH.

### Transfection of Odonto-MSC with siRNA for *NEFL* knockdown

Odonto-MSC at 80% confluence were transfected with 50 pmol *NEFL* siRNA using Lipofectamine RNAiMAX. As a control, Odonto-MSC and Rescued-MSC were transfected with 50 pmol scramble-siRNA. Transfected Odonto-MSC were differentiated into Scramble-Odonto-OD-like cells (Scr-Odonto) or si-NEFL-OD-like cells. Transfected Rescued-MSC were differentiated into Scramble-Rescued-OD-like cells (Scr-Rescued).

### Transfection of Rescued-MSC with vectors for *NEFL* overexpression

For transient transfection, Rescued-MSC at 80% confluence were transfected with 2 μg pRP[Exp]-EGFP/Puro-CMV>hNEFL[NM_006158.5] or pRP[Exp]-EGFP/Puro-CMV>ORF_Stuffer using Lipofectamine 2000 reagent. After 24 h of transfection, each cell was differentiated into Rescued-OD-like cells (NEFL overexpression or mock).

### Statistical analysis

The results are presented as the mean ± SEM or percentage. Differences were tested using ANOVA followed by Tukey’s HSD post hoc test, as appropriate, using JMP software (version 17.0; SAS Institute Inc.). Statistical significance was set at *p* values < 0.05.

## Results

### OD-like cells differentiated from healthy human iPSCs exhibit distinctive cell processes and increased expression of cell polarity markers

**Fig. 1:**
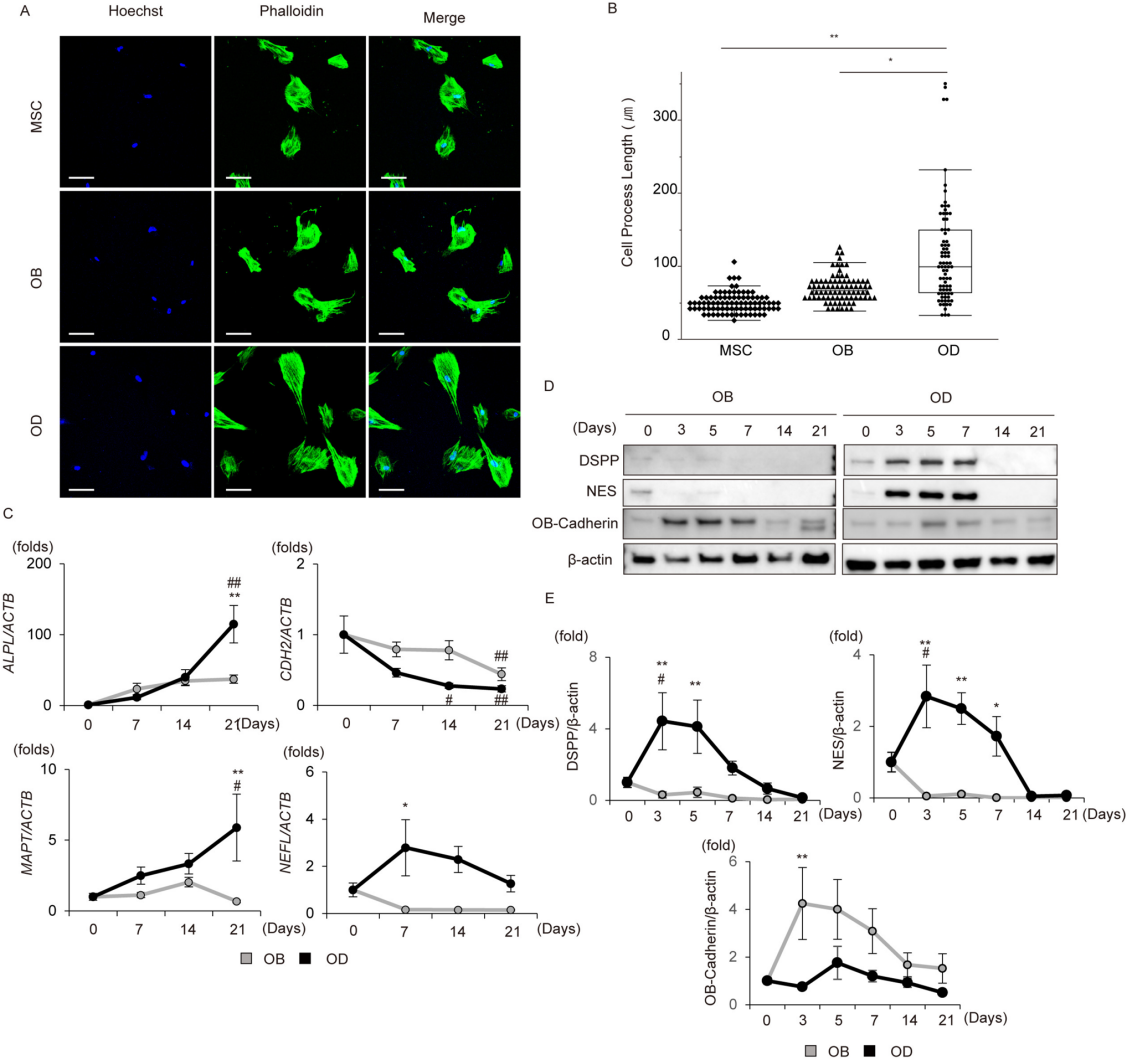
OD-like cells differentiated from healthy human iPSCs exhibit distinctive cell processes and increased expression of cell process formation and OD differentiation markers. **A** Immunofluorescence staining of MSC, OB-like cells, and OD-like cells on day 4 of culture. MSC, OB-like cells, and OD-like cells were cultured in maintenance medium or respective induction medium. Nuclei were stained with Hoechst (blue), and F-actin was stained with phalloidin (green). Scale bars, 100 μm. **B** Measurement of cell process length in MSC. OB-like cells and OD-like cells. The distance from the leading edge to the nucleus was measured in MSC at day 0, OB-like cells and OD-like cells at day 4. *n* = 81–85. ** *p* < 0.01. **C** RT-qPCR of *ALPL*, *CDH2*, *MAPT*, and *NEFL*. Gene expression levels are presented relative to MSC (day 0). Internal control: *ACTB*. **D** Western blotting of OD differentiation markers in OB-like cells and OD-like cells. **E** Quantitative analysis of protein expression of DSPP, NES, and OB-Cadherin. Internal control: β-actin. Data are expressed as mean ± SEM (*n* = 9). *p < 0.05, **p < 0.01 compared with OB-like cells at the same time point; #*p* < 0.05, ##*p* < 0.01 compared to MSC at day 0. *MSC* mesenchymal stem cells, *OB* OB-like cells, *OD* OD-like cell, *DSPP* dentin sialophosphoprotein, *NES* nestin, *OB-Cadherin* osteoblast-cadherin

We created OD-like cells and OB-like cells which were differentiated from NCC-induced MSC from healthy human iPSC using a method from previous reports [22, 30]. OD-like cells differentiated from healthy human iPSCs showed elongation of the cell process in one direction (Fig. 1A). First, we examined the phenotype of OD-like cells compared to that of MSC at day 0. The length of cell processes in OD-like cells was significantly longer than that in MSC at day 0 (Fig. 1B). Gene expression analyses over 21 days revealed OD-like cells differentiation (Fig. 1C). Reverse transcription quantitative polymerase chain reaction (RT-qPCR) analysis showed that the expression of *ALPL* significantly increased in OD-like cells at day 21 than that at day 0 (Fig. 1C). In addition, the expression of *Cadherin 2* (*CDH2*), which increases in early stage of OD differentiation and then decreases in its late stage, decreased in OD-like cells from day 14 to day 21 compared to that in day 0. The expression levels of *MAPT*, which is known cell polarity marker, were higher in OD-like cells at day 21 compared to those in day 0. Western blotting showed that the expression of OD differentiation markers, DSPP and NES, increased in OD-like cells from day 3 to day 7 compared to that in day 0 (Fig. 1D and E). The ALP activity and mineralization ability of OD-like cells increased over time (Supplementary Fig. 1). Mineralization in the OD-like cells was detected after 14 days of OD-like cells differentiation. Quantitative analysis of mineralization indicated a clear increase in calcium deposition in OD-like cells.

We examined characteristics of OD-like cells compared with those of OB-like cells. OD-like cells showed elongation of the cell process in one direction compared to OB-like cells (Fig. 1A). The length of cell processes in OD-like cells was significantly longer than that in OB-like cells (Fig. 1B). In addition, the expression levels of *MAPT* and *NEFL* were higher in OD-like cells than those in OB-like cells (Fig. 1C). The expression of DSPP and NES increased in OD-like cells from day 3 to day 7 compared to that in OB-like cells (Fig. 1D and E). In contrast, OB-cadherin expression was higher in OB-like cells than that in OD-like cells. We confirmed differentiation from MSC to OB-like cells because OB-like cells have mineralization ability and expression of OB-cadherin, a differentiated osteoblast marker. Our results demonstrated characteristics of OD-like cells from human iPSC via NCC in vitro and distinction from OB-like cells.

### Isogenic HPP-iPSCs with the same genetic background showed ALP activity linking to the HPP genotype

**Fig. 2:**
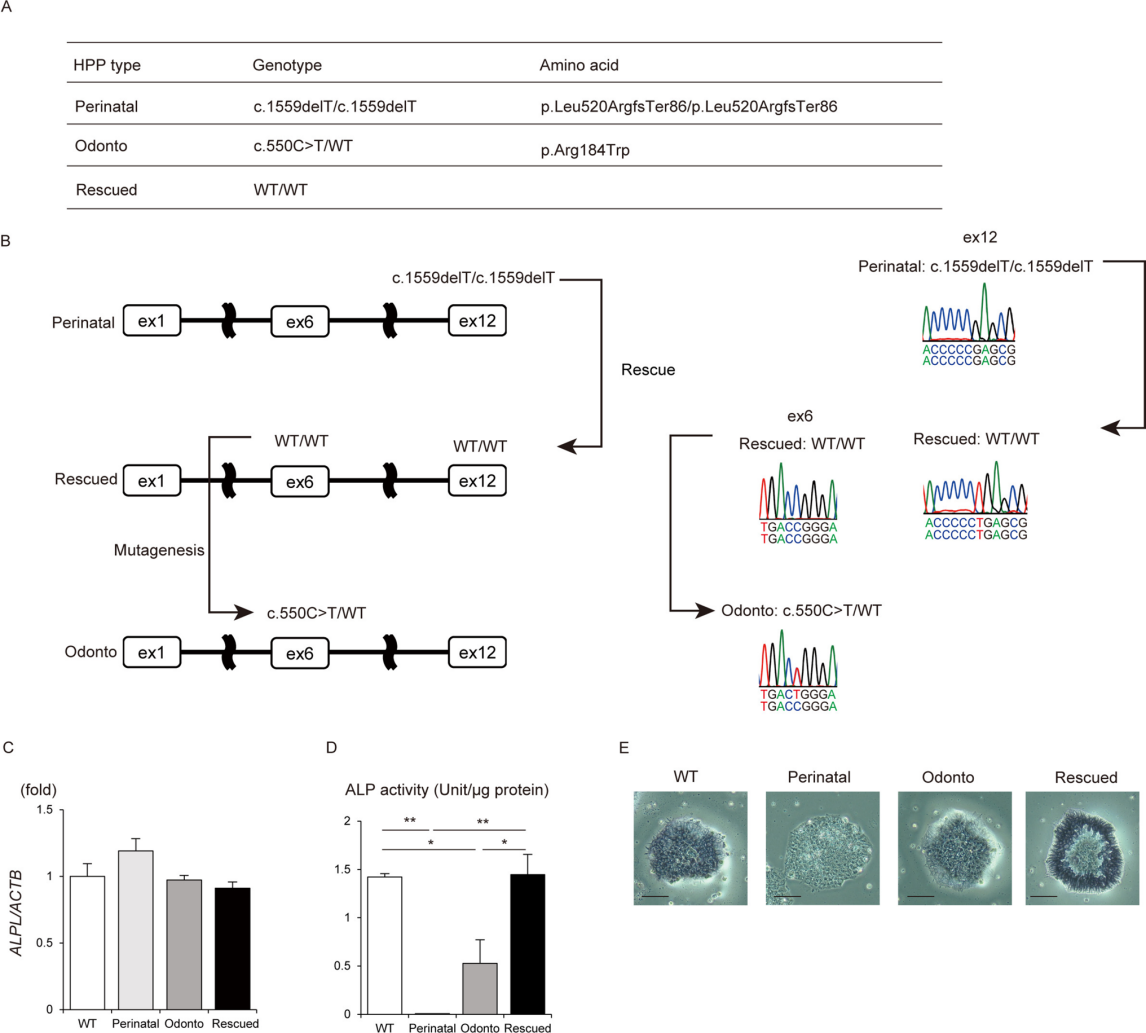
Isogenic HPP-iPSCs with the same genetic background showed ALP activity linking to the HPP genotype. **A** List of HPP types after gene editing. **B** Strategy of gene editing with CRISPR-Cas9 and Prime editing. We generated HPP-iPSCs from a patient with perinatal severe HPP (c.1559delT/c.1559delT: Perinatal) and genetically rescued Perinatal-iPSCs by repairing both alleles using CRISPR-Cas9 (WT/WT: Rescued). In addition, we created an odontohypophosphatasia model (c.550C>T/WT: Odonto) from the Rescued-iPSCs using prime editing. DNA sequence analysis of perinatal-, Odonto-, and Rescued-iPSCs. **C** RT-qPCR of *ALPL* in perinatal-, Odonto- and Rescued-iPSCs relative to WT-iPSCs. Internal control: *ACTB*. **D** ALP activity of WT-, perinatal-, Odonto-, and Rescued-iPSCs. Data are expressed as mean ± SEM. *n* = 3. **E** ALP staining of WT-, perinatal-, Odonto-, and Rescued-iPSCs. Scale bars, 100 μm. *p < 0.05, **p < 0.01. *ex* exon, *WT* wild type

To elucidate the pathophysiology of ODs in patients with HPP caused by pathogenic variants in *ALPL* and investigate the difference in the same genetic background (isogenic), gene editing was performed in iPSC derived from Perinatal (Perinatal-iPSC) [26]. Using CRISPR-Cas9 in Perinatal-iPSC, we succeeded in creating rescued HPP-iPSC (Rescued-iPSC) where the pathogenic variant was repaired (Fig. 2A and B). In addition, we created an Odonto-iPSC model by introducing a single-base substitution of c.550C>T (p.Arg184Trp) in Rescued-iPSC using prime editing (Fig. 2A and B). The monoallelic heterozygous for c.550C>T (p.Arg184Trp) in *ALPL* has been previously reported in patients with Odonto and is registered as a pathogenic variant [2, 31]. RT-qPCR analysis revealed no significant differences in the expression of *ALPL* between WT-, Perinatal-, Odonto- and Rescued-iPSC (Fig. 2C). The ALP activity significantly increased in Rescued-iPSC (Fig. 2D and E), and it improved to the same level as that in WT-iPSC. The ALP activity in Odonto-iPSC was slightly higher than that in Perinatal-iPSC, but lower than that in Rescued-iPSC (Fig. 2D and E).

### Perinatal-OD-like cells and Odonto-OD-like cells showed decreased expression of OD differentiation markers and *MAPT* and mineralization ability

**Fig. 3:**
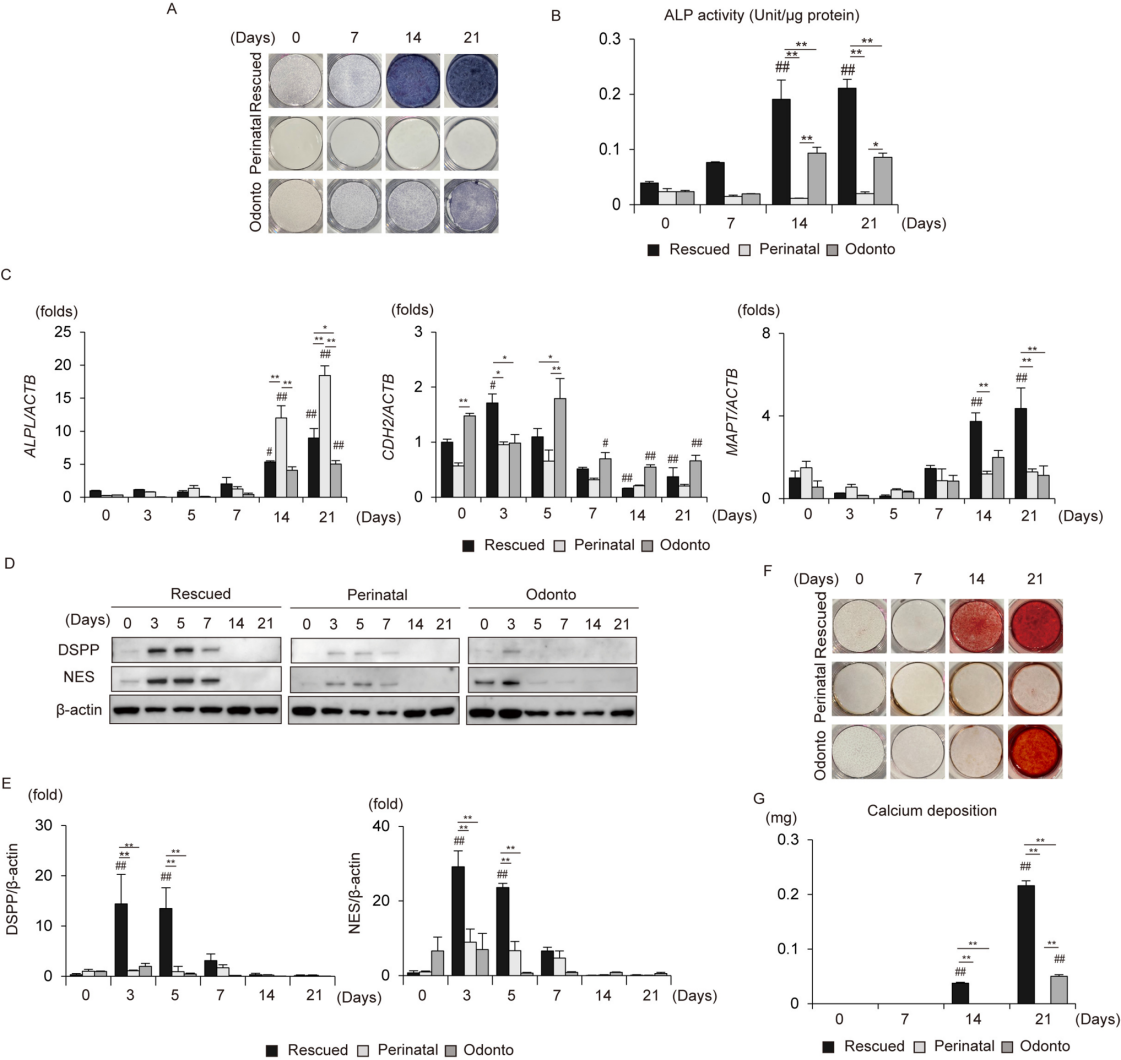
Perinatal-OD-like cells and Odonto-OD-like cells showed decreased expression of OD differentiation markers and *MAPT* and mineralization ability. **A** ALP staining of Rescued-, Perinatal- and Odonto-OD-like cells. **B** ALP activity of Rescued-, Perinatal- and Odonto-OD-like cells. **C** RT-qPCR of *ALPL*, *CDH2*, and *MAPT* in Rescued-, Perinatal- and Odonto-OD-like cells. The expression level of each gene is shown as a value relative to Rescued-OD-like cells at day 0. Internal control: *ACTB*. **D** Western blotting of ODs differentiation markers during OD-like cell differentiation culture. **E** Quantitative analysis of protein expression of DSPP and NES in Rescued-, Perinatal- and Odonto-OD-like cells. Internal control: β-actin. **F** Alizarin red staining of Rescued-, Perinatal- and Odonto-OD-like cells. **G** The amount of calcium deposition by Rescued-, Perinatal- and Odonto-OD-like cells. Data are expressed as mean ± SEM. *n* = 3. *p < 0.05, **p < 0.01 compared with each OD-like cells at the same time points; #*p* < 0.05, ##*p* < 0.01 compared to day 0 of each cell lines; *MSC* mesenchymal stem cell, *DSPP* dentin sialophosphoprotein,* NES* nestin

We created HPP-OD-like cells from perinatal-, Odonto- and Rescued-iPSC using an OD-like cells differentiation system. We used Rescued-iPSC as an isogenic control. We examined OD differentiation and mineralization to investigate the cause of shortened cell process elongation in Perinatal-OD-like cells and Odonto-OD-like cells. The ALP activity significantly increased in Rescued-OD-like cells (Fig. 3A and B), and it showed a slight increase in Odonto-OD-like cells as compared to that in Perinatal-OD-like cells but was lower than that in Rescued-OD-like cells (Fig. 3A and B). The expression of *CDH2* in Odonto-OD-like cells and Rescued-OD-like cells at day 14 and 21 decreased compared to that at day 0 of each line, but no significant difference was observed in Perinatal-OD-like cells between day 0 and day 21 (Fig. 3C). The expression of *MAPT* in Perinatal- and Odonto-OD-like cells was lower than that in Rescued-OD-like cells (Fig. 3C), and the expression of DSPP and NES decreased in Odonto- and Perinatal-OD-like cells on days 3 and 5 of culture compared to that in Rescue-OD-like cells (Fig. 3D and E). Alizarin red staining and calcium deposition measurement showed a significant increase in the mineralization ability in Rescued- and Odonto-OD-like cells from days 14 and 21 of culture, respectively, indicating a delay of mineralization in Odonto-OD-like cells. Perinatal-OD-like cells did not show staining or calcium deposition, even on day 21 (Fig. 3F and G). Our results in Perinatal- and Odonto-OD-like cells suggest that the expression of OD differentiation markers and *MAPT* and mineralization ability were reduced in association with decreased ALP activity.

### Although the elongation of cell process in Perinatal-OD-like cells and Odonto-OD-like cells was short, the expression of *NEFL* in Odonto-OD-like cells was significantly enhanced compared to that in Perinatal-OD-like cells

**Fig. 4:**
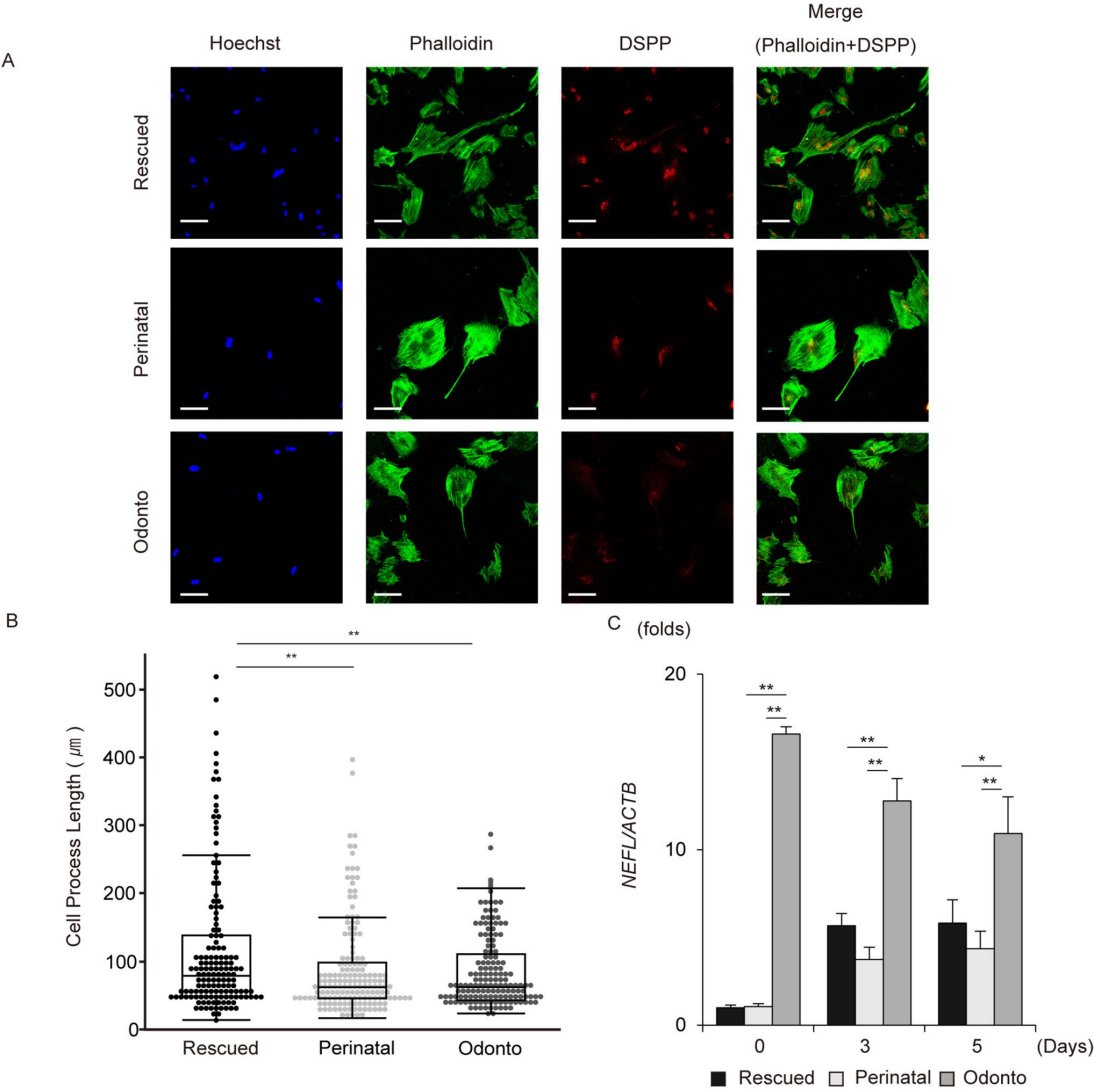
Although the elongation of cell process in Perinatal-OD-like cells and Odonto-OD-like cells was short, the expression of *NEFL* in Odonto-OD-like cells significantly was enhanced compared to that in Perinatal-OD-like cells. **A** Immunofluorescence staining of Rescued-OD-like cells, Perinatal-OD-like cells and Odonto-OD-like cells on day 4 of culture. Nuclei were stained with Hoechst (blue); F-actin, phalloidin (green); DSPP (red). Scale bars, 100 μm. **B** Measurement of cell process length in Perinatal-OD-like cells, Odonto-OD-like cells, and Rescued-OD-like cells. The distance from the leading edge to the nucleus was measured in OD-like cells on day 4. *n* = 146–166. **p < 0.01. C. RT-qPCR of *NEFL* expression in Rescued-, Perinatal- and Odonto-OD-like cells. Internal control: *ACTB*. *n* = 9. *p < 0.05, **p < 0.01 compared with each OD-like cells at the same time point. Data are expressed as mean ± SEM. *MSC* mesenchymal stem cells

We investigated the elongation of the cell processes in OD-like cells. All HPP-OD-like cells elongated the cell processes in one direction (Fig. 4A). The length of cell processes in Perinatal- and Odonto-OD-like cells was shorter than that in Rescued-OD-like cells (Fig. 4B). In contrast, the expression of *NEFL* was enhanced in Odonto-OD-like cells compared to that in Perinatal- and Rescued-OD-like cells (Fig. 4C). These results suggest that the enhanced expression of *NEFL* might bring about a specific pathophysiology in Odonto-OD-like cells compared to Perinatal-OD-like cells. To explore why *NEFL* is upregulated in Odonto-OD-like cells, we overexpressed six Odonto-HPP-associated *ALPL* variants including c.211 C>T (p.Arg71Cys), c.215 T>C (p.Ile72Thr), c.323 C>T (p.Pro108Leu), c.550 C>T (p.Arg184Trp), c.1285 G>A (p.Glu429Lys), and c.1375 G>T (p.Val459Leu) in HEK293 cells. However, we did not detect variant-specific differences in *NEFL* expression in this transient overexpression experiments (Supplementary Fig. 2). We also evaluate predicted structural change in these variants using 3D modeling and found no major structural deviations from WT based on root mean square deviation (RMSD) (Supplementary Fig. 3, 4; Supplementary Table 5).

### *NEFL* downregulation promotes elongation of Odonto-OD-like cell process, whereas* NEFL* overexpression shortens them

**Fig. 5:**
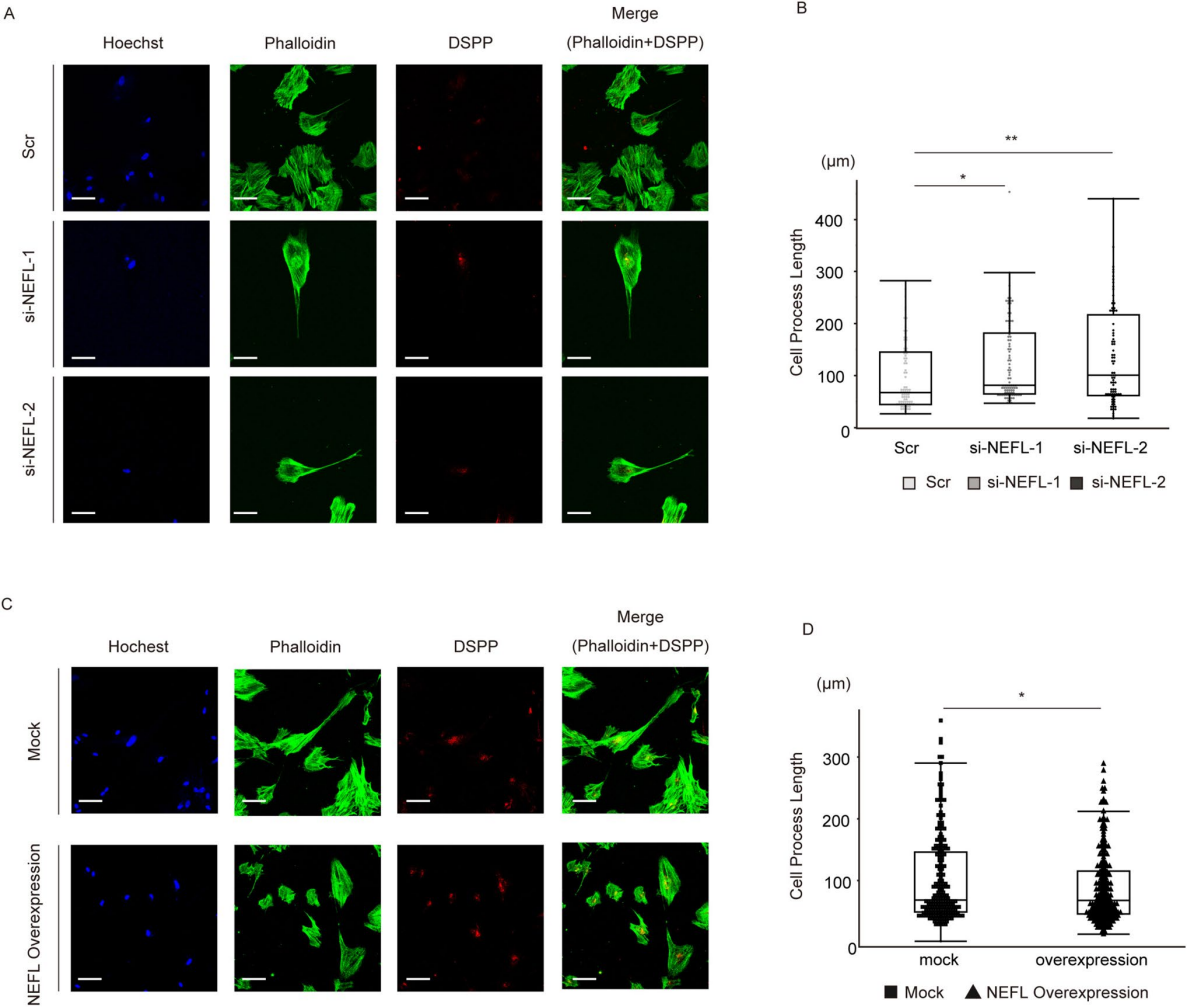
*NEFL* expression level in Odonto-OD-like cells determined cell process elongation. **A** Immunofluorescence staining of Scr- and si-NEFL-OD-like cells on day 4. Nuclei were stained with Hoechst (blue); F-actin, phalloidin (green); DSPP (red). Scale bars, 100 μm. **B** Measurement of odontoblastic process length. The distance from the leading edge to the nucleus was measured in all cells on day 4. *n* = 80–93. **C** Immunofluorescence staining of Mock-OD-like cells and NEFL-overexpression-OD-like cells on day 4. Nuclei were stained with Hoechst (blue); F-actin, phalloidin (green); DSPP (red). Scale bars, 100 μm. **D** Measurement of odontoblastic process length. The distance from the leading edge to the nucleus was measured in all cells on day 4. *n* = 206–216. *p < 0.05, **p < 0.01 compared with each OD-like cells at the same time point. Data are expressed as mean ± SEM. *siRNA* small interfering RNA, *Scr* scramble-siRNA

To investigate the pathological effects of *NEFL* in Odonto-OD-like cells, *NEFL* was knocked down in Odonto-MSC using small interfering RNA (siRNA) before differentiation into OD-like cells. Transfected Odonto-MSC differentiated into Odonto-OD-like cells (si-NEFL-OD-like cells). As a control, Odonto-MSC was transfected with scrambled siRNAs and differentiated into Odonto-OD-like cells (Scr-Odonto-OD-like cells). *NEFL* expression was lower in si-NEFL-OD-like cells than that in Scr-OD-like cells (Supplementary Fig. 5). Immunofluorescence staining showed that both si-NEFL-OD-like cells and Scr-OD-like cells elongated the cell processes in one direction, indicating proper cell polarity (Fig. 5A and B). However, the length of the cell processes in si-NEFL-OD-like cells was longer than that in Scr-OD-like cells. *NEFL* knockdown in Odonto-OD-like cells improved elongation of the cell processes. Moreover, the ALP activity remained low after *NEFL* knockdown (Supplementary Fig. 6). Our results suggest that the abnormal cell morphology of ODs in patients with Odonto is affected by the enhanced expression of *NEFL* in Odonto-OD-like cells, and the defect in cell process elongation is not directly caused by low ALP levels. Next, we performed overexpression of *NEFL* in OD-like cells (Supplementary Fig. 7). Although immunofluorescence staining showed that both NEFL-overexpression-OD-like cells and Mock-OD-like cells elongated the cell processes in one direction, the length of the cell processes in NEFL-overexpression-OD-like cells was shorter than that in Mock-OD-like cells (Fig. 5C and D), indicating that *NEFL* overexpression in Rescued-OD-like cells impaired elongation of the cell processes. Our results suggest that enhanced *NEFL* expression negatively regulates elongation of the odontoblastic cell processes in patients with HPP.

## Discussion

In the present study, we used iPSCs derived from Perinatal and Odonto to investigate cellular mechanisms that may underlie dentin-related abnormalities and dental manifestations across HPP subtypes and the cause of dental manifestations in HPP. We selected homozygous c.1559delT variant in *ALPL*, which was found in most Japanese patients with Perinatal, and heterozygous c.550C>T (p. Arg184Trp) variant in *ALPL* found in our patients with Odonto as the genotype [2]. We demonstrated that *NEFL* was highly expressed, and that its expression level was associated with the pathophysiology of abnormal ODs in patients with Odonto. In addition, the inverse correlation of the *NEFL* expression and cell process elongation was observed. The short elongation of the cell processes in Odonto-OD-like cells was associated with the enhanced expression of *NEFL*, and the elongation of cell processes in Odonto-OD-like cells was restored by the *NEFL* knockdown. Since the cell process elongation plays the role in OD-like cells [23] and, in our odonto-HPP-iPSC model (c.550C>T; p.Arg184Trp), increased *NEFL* expression was associated with shortened odontoblastic processes and modulated process length independently of ALP activity, suggesting that *NEFL* may contribute to the odontoblast morphological phenotype in this setting.

*NEFL* encodes a type IV intermediate filament which functionally maintains neuronal caliber and plays an important role in the intracellular transport of neurotransmitters to axons and dendrites [32]. *NEFL* is found in neurons but has also been observed in dental pulp cells [19]. It has been reported that *NEFL* overexpression causes the accumulation of NEFL proteins around the nucleus, leading to the downregulation of cell polarity such as axonal atrophy [33]. In contrast, neuronal degradation and atrophy occur in neurons with downregulated *NEFL* [34]. Therefore, an adequate expression level of *NEFL* is important for the maintenance of cell process elongation.

Notably, the *NEFL* knockdown was not associated with the recovery of ALP activity, while the knockdown resulted in the recovery of the OD phenotype. These results suggest that the mechanism by which the enhanced expression of *NEFL* may contribute to the tooth phenotype is distinct from the mechanism underlying low ALP activity. Further studies are warranted to elucidate the precise mechanism involved [9].

The mineralization ability in Perinatal-OD-like cells and Odonto-OD-like cells showed a correlation with ALP activity. Although bone can restore the skeleton by remodeling, teeth with ODs are not remodeled once they are formed, leading to the permanent effects [35]. Therefore, hypomineralization of mantle dentin in deciduous teeth in both Perinatal and Odonto was persistent, and this may explain why the deterioration of the cementum-forming scaffold is deteriorated both in perinatal and odonto type of HPP.

We verified the OD phenotype by detecting the simultaneous expression of DSPP and NES, as previously reported [36]. In addition, the decreased expression of *CDH2* in WT-OD is consistent with previous reports [37, 38]. According to previous reports, DSPP is primarily expressed in ODs and to a lesser extent in OBs [39, 40]. DSPP becomes a functional protein by hydrolysis into DSP and DPP. DSP regulates the initiation of dentin mineralization, and DPP regulates the maturation of dentin mineralization [41]. The hydrolysis of DSPP into DSP and DPP is likely to be the reason why DSPP was not detected in WT-OD on day 14 and after. In addition, it is known that NES is only expressed in functional ODs within unmineralized dentin and not within mineralized one [42]. Our results showed that NES expression in WT-OD-like cells was detected on days 3, 5 and 7 of culture when mineralization did not start yet, and that NES expression was not observed on days 14 and 21 when mineralization occurred, which is consistent with the previous report [42]. Our OD-like cells differentiation protocol differs from previously reported direct differentiation protocols [22, 23], in that it proceeds via NCC, reflecting the developmental origin of odontoblasts and providing more consistent differentiation marker expression in our hands. Our differentiation protocol from human iPSC into OD-like cells via NCC approximates the physiological differentiation. However, we have not demonstrated our OD-like cells differentiation protocol in vivo.

Our results also showed that OD-like cells differentiated from iPSCs exhibited increased expression of *MAPT* and elongated cell processes in one direction using immunofluorescence staining. MAPT is expressed in terminally differentiated OD, and it plays an important role in determining the features of cell polarity and morphogenesis of cell processes in OD by regulating microtubule organization [15]. The cell processes in ODs are mainly involved in collagen secretion and mineralization in dentin [16, 17]. Therefore, elongation of the OD cell process is essential for dentin formation. Furthermore, the expression of *MAPT* and elongation of the cell processes were impaired in Perinatal- and Odonto-OD-like cells. These results suggest that cell polarity characterized by elongation of cell processes in one direction is an essential event that requires in normal OD function.

This study has potential limitations. We have proven the characterization of OD-like cells in vitro, but not in vivo, and have not shown OD-like cells differentiation in time-lapse images. The analysis of OD characterizations and transplantation of OD-like cells under the renal capsule is required in vivo in the future. In addition, we could not prove why *NEFL* expression was increased in odonto-OD-like cells patients with Odonto. We used single-donor iPSC derived from a patient with HPP. The number of samples of Perinatal and Odonto used in our study was one for each phenotype. In the future, samples from patients with other variants should be assessed for further comparison. However, our results demonstrated that altered NEFL expressions were associated with cell process elongation in OD at least with heterozygous c.550C>T (p. Arg184Trp) in *ALPL* gene. Importantly, our odonto-HPP findings are based on a single engineered odonto genotype (c.550C>T; p. Arg184Trp) in one genetic background; therefore, these results should be interpreted as hypothesis-generating rather than generalized to all odontohypophosphatasia.

In conclusion, the expression of OD differentiation markers and *MAPT*, and mineralization ability decreased in HPP-OD-like cells via reduction of ALP activity. Notably, the enhanced expression of *NEFL* in Odonto-OD-like cells suggests a specific pathophysiology of ODs in patients with Odonto, which may be associated with impaired elongation of cell processes, without being linked to the recovery of ALP activity compared with Perinatal-OD-like cells and Rescued-OD-like cells. These findings suggest that *NEFL* may affect the pathophysiology of abnormal ODs in patients with Odonto and might provide clues for the development of tooth-specific treatments for patients with HPP.

## Supplementary Information

Below is the link to the electronic supplementary material.

Supplementary file1 (DOCX 1707 kb)

## Data Availability

No data were used for the research described in the article.
