# Supplementary material for: NEFL is associated with inhibition of odontoblastic process in odontohypophosphatasia
Source: J Bone Miner Metab. 2026 Mar 14;44(3):429–41. doi: 10.1007/s00774-026-01703-5 (PMC13246916; doi:10.1007/s00774-026-01703-5)
Supplement: Supplementary file 1 — Supplementary file1 (DOCX 1707 kb) [file 774_2026_1703_MOESM1_ESM.docx]

**NEFL is** **associated with inhibition of odontoblastic process in odontohypophosphatasia**

Akira Nozoe, Yasuhisa Ohata, Makoto Fujiwara, Kenichi Yamamoto, Toshihiko Nambara, Chiho Nakano, Kazuaki Miyagawa, Mikihiko Kogo, Takeshi Taketani, Takuo Kubota, Yasuji Kitabatake, Susumu Tanaka, Keiichi Ozono

**Supplemental materials and methods**

**Construction of expression vectors**

Expression vectors encoding human tissue-nonspecific alkaline phosphatase (ALPL) wild-type (WT) and mutant proteins were constructed using the mammalian expression vector pcDNA3.1. The ALPL WT expression vector had been generated previously* and was used as a template for mutagenesis. Six ALPL variants associated with odontohypophosphatasia (odonto-HPP) (c.550C>T, c.211C>T, c.215T>C, c.323C>T, c.1285G>A, and c.1375G>T) were selected based on the Tissue-nonspecific Alkaline Phosphatase Gene Mutation Database (https://www.sesep.uvsq.fr/03_hypo_mutations.php). In addition, one variant associated with perinatal severe hypophosphatasia (c.1559delT) was included. Site-directed mutagenesis was performed using the PrimeSTAR® Mutagenesis Basal Kit (TaKaRa) according to the manufacturer’s instructions to introduce each mutation. Following mutagenesis, plasmids were verified by Sanger sequencing to confirm the presence of the intended mutations. In total, nine expression vectors were generated and used for subsequent experiments, including the ALPL WT expression vector, seven mutant expression vectors (six odonto-HPP–associated variants and one perinatal severe variant), and an empty vector (pcDNA3.1).

*Cai G, Michigami T, Yamamoto T, Yasui N, Satomura K, Yamagata M, Shima M, Nakajima S, Mushiake S, Okada S, Ozono K. Analysis of localization of mutated tissue-nonspecific alkaline phosphatase proteins associated with neonatal hypophosphatasia using green fluorescent protein chimeras. J Clin Endocrinol Metab. 1998 Nov;83(11):3936-42. doi: 10.1210/jcem.83.11.5267. PMID: 9814472.

**Transfection and RT-qPCR**

HEK293 cells were cultured in Dulbecco’s Modified Eagle’s medium (DMEM) supplemented with 10% fetal bovine serum and 1% antibiotics at 37°C in a humidified atmosphere containing 5% CO₂. Cells were seeded in 6-well plates and transfected at ~90% confluence with ALPL WT, ALPL variant, or empty pcDNA3.1 plasmids using Lipofectamine 3000 (Invitrogen) following the manufacturer’s protocol. Seventy-two hours after transfection, total RNA was extracted using the RNeasy Mini Kit, and cDNA was synthesized using ReverTra Ace qPCR RT Master Mix. Quantitative PCR was performed using THUNDERBIRD SYBR qPCR Mix on a QuantStudio 7 Flex Real-time PCR System with gene-specific primers. Relative expression was calculated by the 2⁻ΔΔCt method and normalized to GAPDH. Statistical analyses were performed as described in the figure legends. P values < 0.05 were considered statistically significant, and multiple-comparison correction was applied where appropriate.

**Structure prediction and structural analysis**

Three-dimensional structural models of human tissue-nonspecific alkaline phosphatase (ALPL) were generated using AlphaFold2 (https://colab.research.google.com/github/sokrypton/ColabFold/blob/main/AlphaFold2.ipynb). For the wild-type (WT) condition, homodimeric WT models were constructed, whereas for each variant, heterodimeric models consisting of one WT subunit and one variant subunit were generated. Protein sequences corresponding to the WT and each missense variant were individually submitted to AlphaFold2, and the top-ranked predicted models were used for subsequent analyses. Heterodimeric models were generated by combining wild-type and variant monomeric models in a configuration consistent with the predicted dimeric arrangement.

All molecular graphics and figures were generated using UCSF ChimeraX (version 1.10).

Structural similarity between the wild-type (WT) and each variant model was assessed by structural superposition using the MatchMaker tool in UCSF ChimeraX, and RMSD values displayed by the software were used for comparison.

**

Supplementary Table 1. List of antibodies for Immunofluorescence staining and Western Blotting

| Name | Resource | Catalog Number |
| --- | --- | --- |
| DSPP (LFMb-21) antibody | Santa Cruz Biotechnology | sc-73632, RRID:[AB_2230660](http://antibodyregistry.org/AB_2230660) |
| Nestin (10C2) Mouse mAb antibody | Cell Signaling Technology | 10c2, RRID:[AB_2799037](https://antibodyregistry.org/search.php?q=AB_2799037) |
| OB-Cadherin (P707) Antibody | Cell Signaling Technology | 4442, RRID:[AB_10547881](https://www.antibodyregistry.org/AB_10547881) |
| Anti-B-Actin HRP-DirecT antibody | MBL International | PM053-7, RRID:[AB_10697035](https://antibodyregistry.org/search.php?q=AB_10697035) |
| Goat Anti-Mouse IgG H&L (Alexa Fluor® 594) antibody | Abcam | ab150116, RRID:[AB_2650601](https://antibodyregistry.org/search.php?q=AB_2650601) |
| Anti-rabbit IgG (H+L), F(ab')2 Fragment (Alexa Fluor(R) 488 Conjugate) | Cell Signaling Technology | 4412S,  RRID: AB_1904025 |
| Anti-Mouse IgG (H+L) Antibody, HRP Conjugated | Promega | W4021, RRID:[AB_430834](https://antibodyregistry.org/search.php?q=AB_430834) |
| Anti-Rabbit IgG (H+L), HRP Conjugate antibody | Promega | W4011, RRID:[AB_430833](https://antibodyregistry.org/search.php?q=AB_430833) |

Supplementary Table 2. The primers used for Reverse transcription quantitative polymerase chain reaction assay

| Gene | Forward primer | Reverse primer |
| --- | --- | --- |
| *ALPL* | 5′-CCTCGTTGACACCTGGAAGAG -3′ | 5′- TTCCGTGCGGTTCCAGA -3′ |
| *CDH2* | 5′- ATTGCCATCCTGCTCTGCAT -3′ | 5′- TGGCTCAAGTCATAGTCCTGG -3′ |
| *NEFL* | 5′- AGCTGGAGGACAAGCAGAAC -3′ | 5′- CGCCTTCCAAGAGTTTCCTGT -3′ |
| *MAPT* | 5′- GACGCTGGCCTGAAAGAATC -3′ | 5′- CTTCTGGGATCTCCGTGTGG -3′ |
| *ACTB* | 5′- TCAAGATCATTGCTCCTCCTGAG -3′ | 5′- ACATCTGCTGGAAGGTGGACA -3′ |
| *GAPDH* | 5′- TGCACCACCAACTGCTTAGC -3′ | 5′- GGCATGGACTGTGGTCATGAG -3′ |

Supplementary Table 3. Sequences used for prime editing

| pegRNA | 5′→3′ |
| --- | --- |
| Spacer Oligo Top | caccCGTTGTCTGAGTACCAGTCCgtttt |
| Spacer Oligo Reverse | ctctaaaacGGACTGGTACTCAGACAACG |
| Extension Oligo Top | gtgcGCTGACtGGGACTGGTACTCAGAC |
| Extension Oligo Reverse | cgcgGTCTGAGTACCAGTCCCAGTCAGC |
| Scaffold Oligo Top | AGAGCTAGAAATAGCAAGTTAAAATAAGGCTAGTCCGTTATCAACTTGAAAAAGTGGCACCGAGTCG |
| Scaffold Oligo Reverse | GCACCGACTCGGTGCCACTTTTTCAAGTTGATAACGGACTAGCCTTATTTTAACTTGCTATTTCTAG |

Supplementary Table 4. Sequences used for *NEFL* knockdown

| si-NEFL-1 forward | GGACACGAUCAACAAAUUAtt |
| --- | --- |
| si-NEFL-1 reverse | UAAUUUGUUGAUCGUGUCCtg |
| si-NEFL-2 forward | GGCACGAUACCUAAAAGAAtt |
| si-NEFL-2 reverse | UUCUUUUAGGUAUCGUGCCat |

Supplementary Table 5. ALPL Nucleotide Variants, Corresponding Amino Acid Changes, and Structural Analysis

| Nucleotide Variants | Amino Acid Variants | Residual activity (%) (reported) | Residual activity (%) (this study) | RMSD  (Variant/WT vs WT/WT） | Dominant-negative effect (reported), % activity (WT+variant / WT+WT) |
| --- | --- | --- | --- | --- | --- |
| c.211 C>T | p.Arg71Cys (R71C) | 0.0%^2^ 7.7%^3^ | 0.0% | 0.231 | 35％^4^  33.2％^3^ |
| c.215 T>C | p.Ile72Thr (I72T) | 5.1%^4^ | 0.0% | 0.198 | 40.2％^4^ |
| c.323 C>T | p.Pro108Leu (P108L) | 1.9%^4^ | 0.0% | 0.230 | 24.0％^4^ |
| c.550 C>T | p.Arg184Trp (R184W) | 0.6%^5^ 0.0%^6^ 3.1%^3^ 0.6%^7^ | 0.0% | 0.229 | 36.7％^5^  50.0％^6^  44.1%^3^ |
| c.1285 G>A | p.Glu429Lys (E429K) | 1.3%^4^ | 0.0% | 0.221 | 31.0%^4^ |
| c.1375 G>T | p.Val459Leu (V459L) | 0.0%^4^ | 0.0% | 0.184 | 41.1％^4^ |

Nucleotide variants and corresponding amino acid changes of ALPL variants analyzed in this study are shown. Previously reported residual alkaline phosphatase activity values were obtained from the literature and are presented as reference data. RMSD values were calculated by superposition of AlphaFold2-predicted three-dimensional structures using UCSF ChimeraX. For each variant, structural alignment was performed between the wild-type (WT) homodimer and the WT–variant heterodimer model. Dominant-negative effect values are reported as ALP activity measured in co-expression of WT+variant divided by WT+WT, expressed as a percentage (WT+WT = 100%).


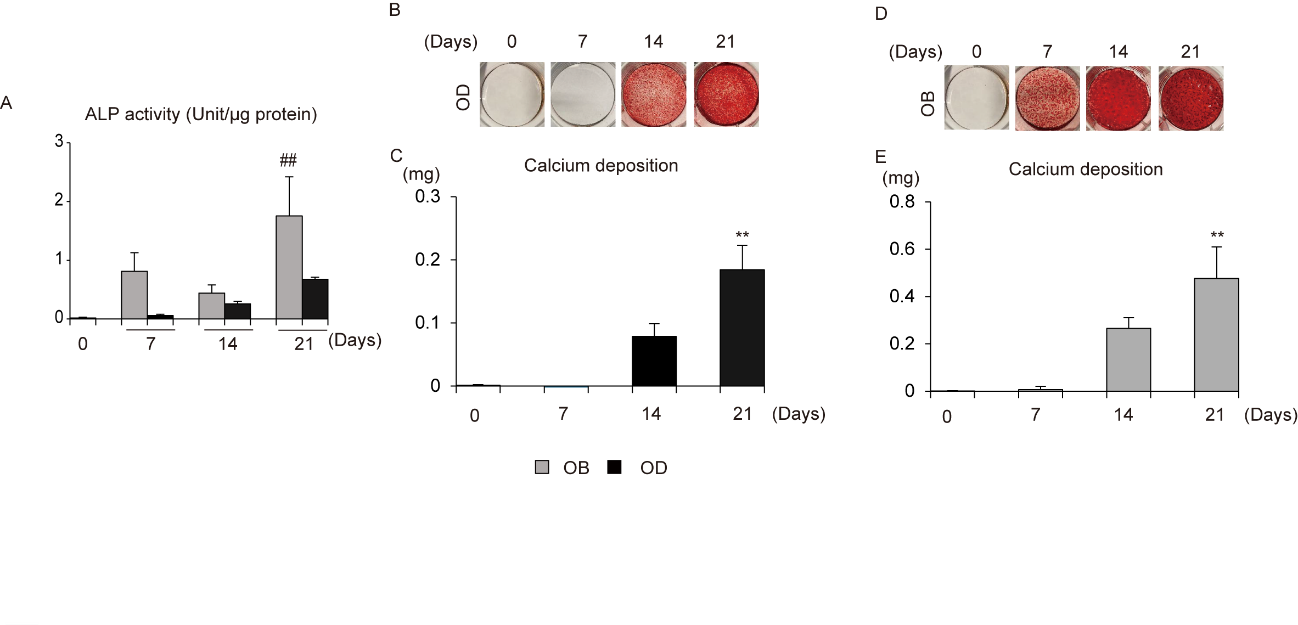


Supplementary Figure 1. ALP activity and mineralization ability in WT-OD-like cells and WT-OB-like cells. A. Alkaline phosphatase activity of WT-OD-like cells and WT-OB-like cells. B and D. Alizarin Red staining in WT-OD-like cells and WT-OB-like cells, respectively. C and E. Quantification of calcium deposition in WT-OD-like cells and WT-OB-like cells, respectively. Data are expressed as mean ± SEM (n = 9). ** *p* < 0.01 compared to day 0. ## *p* < 0.01 compared to MSC at day 0. MSC, mesenchymal stem cells; OD, OD-like cells; OB, OB-like cells.


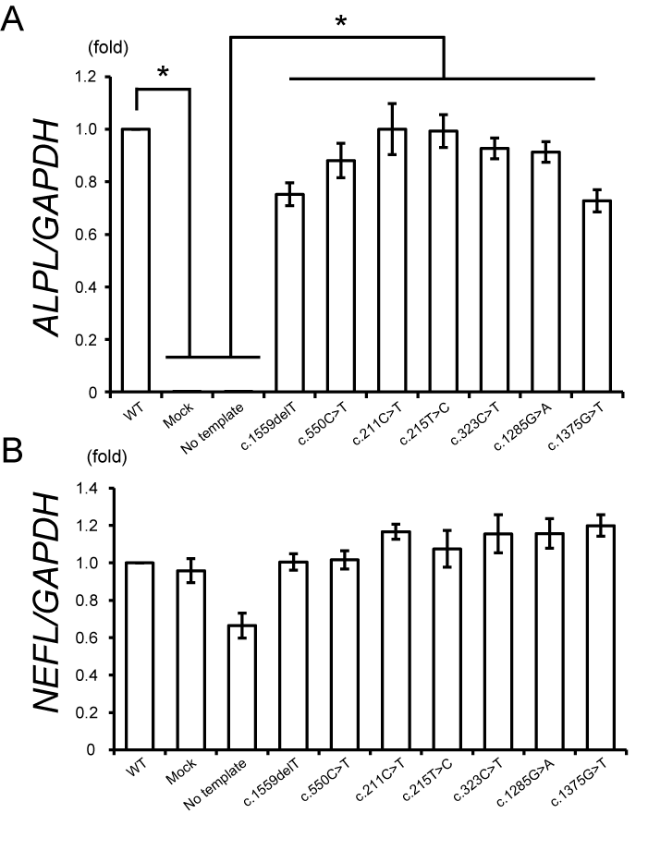


Supplementary Figure 2. qPCR analysis of *ALPL* and *NEFL* expression in HEK293 cells expressing WT ALPL or ALPL variants. HEK293 cells expressing WT ALPL or the indicated ALPL variants were analyzed by RT-qPCR. Empty vector (Mock) and no-template controls were included. *ALPL* (A) and *NEFL* (B) transcript levels were normalized to *GAPDH* and are shown as fold change relative to WT (set to 1; 2⁻ΔΔCt). Data represent three independent experiments; within each experiment, n=3 biological replicates were analyzed (technical duplicates averaged), and error bars indicate SEM. For *ALPL* (A), statistical significance was assessed by one-way ANOVA followed by post hoc comparisons versus Mock and No-template with Holm correction. For *NEFL* (B), statistical significance versus WT was assessed using Holm-corrected comparisons. WT and all variants showed significantly higher *ALPL* expression than Mock and No-template (Holm-corrected p < 0.05), whereas no significant differences in *NEFL* expression were detected relative to WT (Holm-corrected p ≥ 0.05).


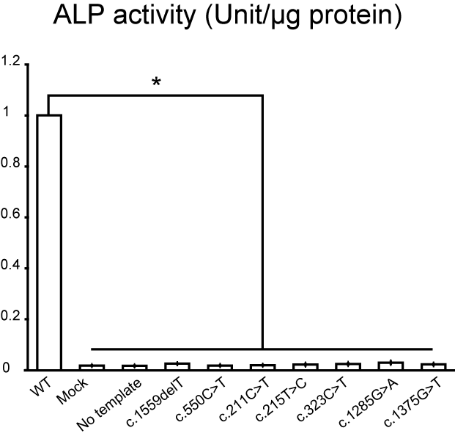


Supplementary Figure 3. Relative ALP activity of WT and ALPL variants ALP activity was measured for WT and each variant in parallel within the same experiment, and the experiment was independently repeated three times. Activities were normalized to the WT measured in the same experiment (WT = 1). Data are presented as mean ± SEM (n = 3 independent experiments). Statistical significance versus WT (WT = 1) was assessed using a two-tailed one-sample t-test. *P < 0.001.
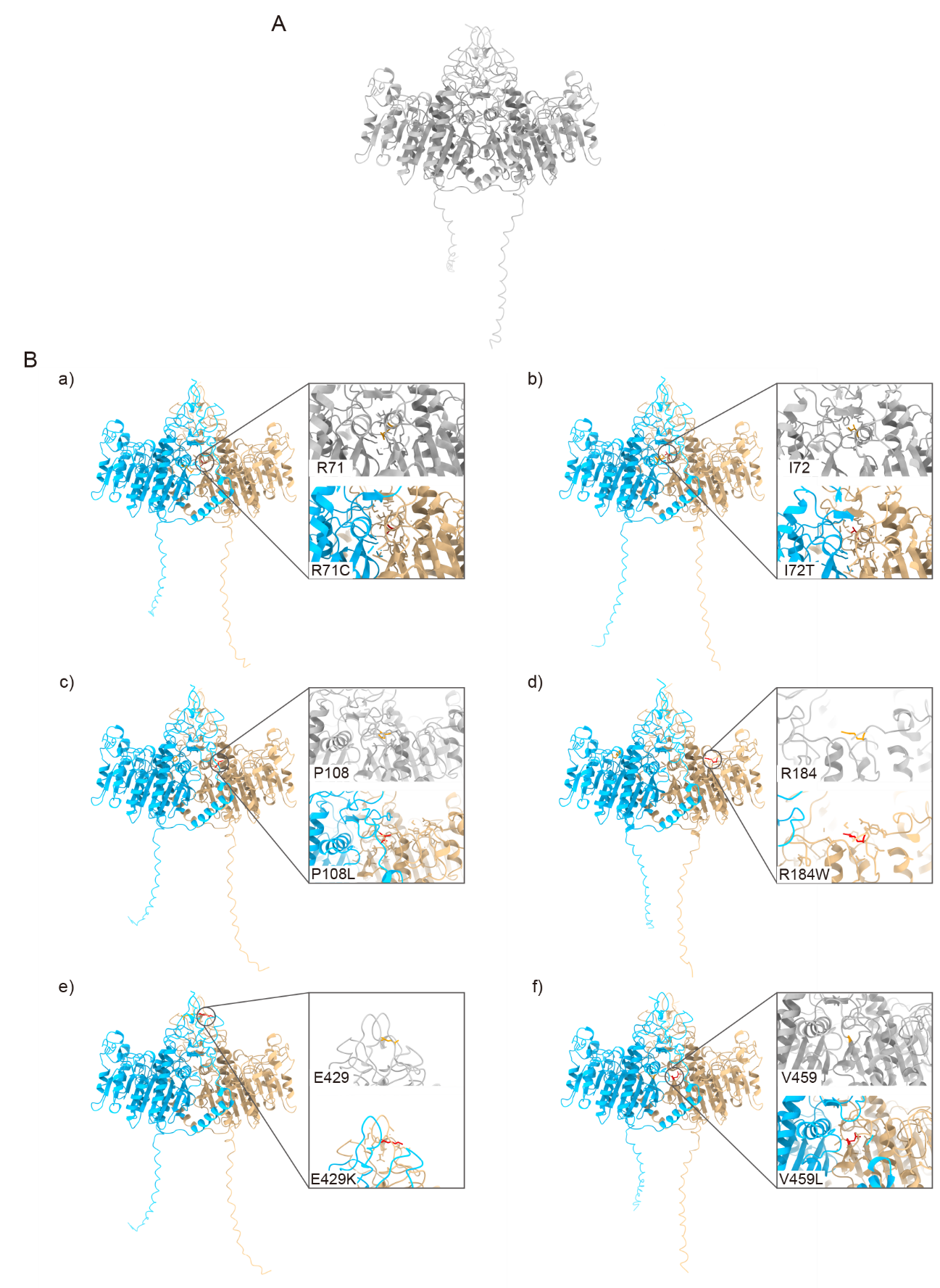


Supplementary Figure 4. Predicted protein structures from AlphaFold2. (A) The homodimeric structure of human ALPL was predicted from the wild-type (WT) amino acid sequence using AlphaFold2 and visualized in UCSF ChimeraX (v1.11). (B) Predicted structures of WT/variant heterodimers for odontohypophosphatasia-associated ALPL missense variants were generated using AlphaFold2 and visualized in ChimeraX (v1.11): (a) R71C (c.211C>T), (b) I72T (c.215T>C), (c) P108L (c.323C>T), (d) R184W (c.550C>T), (e) E429K (c.1285G>A), and (f) V459L (c.1375G>T). In each heterodimer model, the WT subunit is colored deepskyblue and the variant subunit is colored burlywood. For each variant, an overall view is shown alongside enlarged views of the mutation site, with the WT residue shown in the top panel and the variant residue shown in the bottom panel. In the enlarged views, only atoms at the mutated position are displayed as sticks, with WT shown in orange and the variant shown in red.


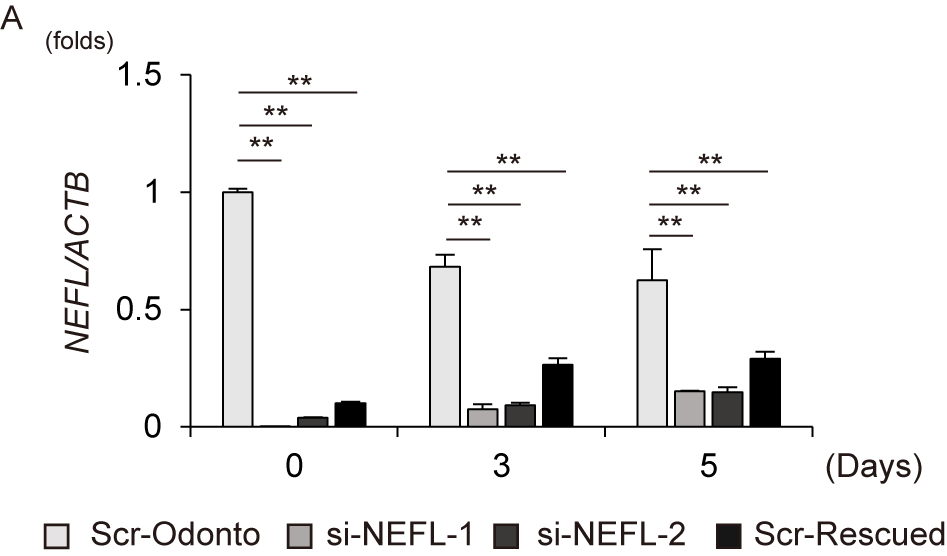
Supplementary Figure 5. Efficiency of *NEFL* knockdown in Odonto-OD-like cells. A. RT-qPCR of *NEFL* expression in Scr-Odonto, si-NEFL-1, si-NEFL-2 and Scr-Rescued. Internal control: ACTB. n = 3. ** *p* < 0.01 compared with each OD-like cells at the same time points. Data are expressed as mean ± SEM. siRNA, small interfering RNA; Scr, scramble siRNA.


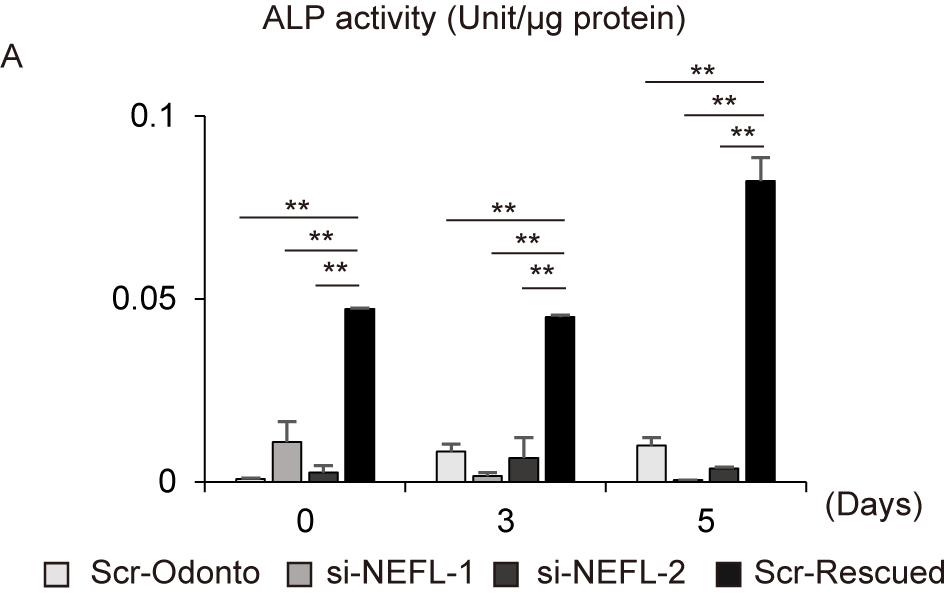
Supplementary Figure 6. ALP activity of Odonto-OD-like cells did not improve by *NEFL* knockdown. A. ALP activity of Scr-Odonto, si-NEFL-1, si-NEFL-2 and Scr-Rescued. n = 3. ** *p* < 0.01 compared with each OD-like cells at the same time points. Data are expressed as mean ± SEM. siRNA, small interfering RNA; Scr, scramble siRNA.


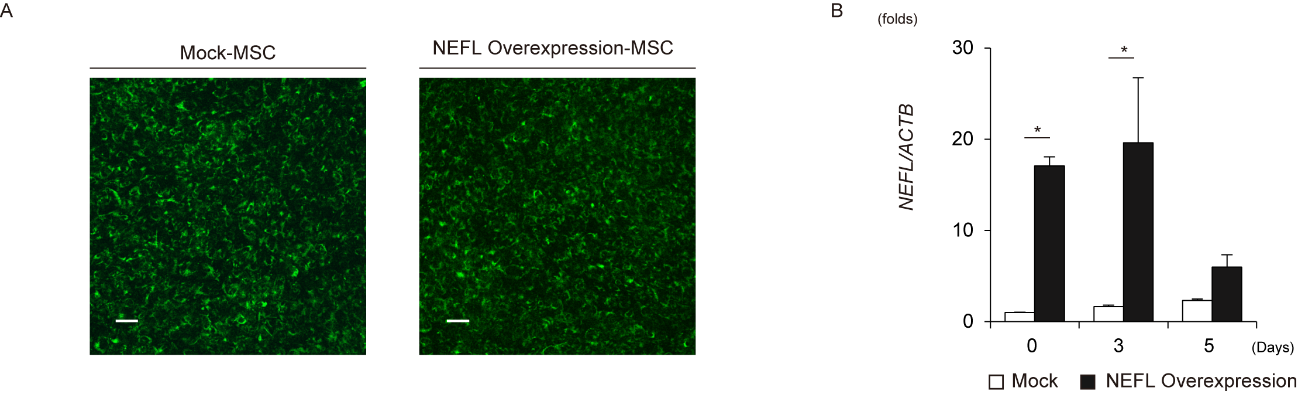
Supplementary Figure 7. Efficiency of *NEFL* overexpression in Rescued-OD-like cells. A. GFP indicates successfully Mock-MSC and NEFL overexpression-MSC after transfection. Rescued-MSC were transduced with pRP[Exp]-EGFP/Puro-CMV>ORF_Stuffer or pRP[Exp]-EGFP/Puro-CMV>hNEFL using the lipofectamine 2000 transfection system. The images were captured by FV3000. Scale bar, 100 μm. B. RT-qPCR of *NEFL* expression in Mock-OD-like cells and NEFL overexpression-OD-like cells. Internal control: ACTB. n = 3. * *p* < 0.05 compared with each OD-like cells at the same time points. Data are expressed as mean ± SEM.

Supplemental reference

1. Jumper J, Evans R, Pritzel A, Green T, Figurnov M, Ronneberger O, Tunyasuvunakool K, Bates R, Žídek A, Potapenko A, Bridgland A, Meyer C, Kohl SAA, Ballard AJ, Cowie A, Romera-Paredes B, Nikolov S, Jain R, Adler J, Back T, Petersen S, Reiman D, Clancy E, Zielinski M, Steinegger M, Pacholska M, Berghammer T, Bodenstein S, Silver D, Vinyals O, Senior AW, Kavukcuoglu K, Kohli P, Hassabis D. Highly accurate protein structure prediction with AlphaFold. Nature. 2021 Aug;596(7873):583-589. doi: 10.1038/s41586-021-03819-2. Epub 2021 Jul 15. PMID: 34265844; PMCID: PMC8371605.

2. Fukushi-Irié M, Ito M, Amaya Y, Amizuka N, Ozawa H, Omura S, Ikehara Y, Oda K. Possible interference between tissue-non-specific alkaline phosphatase with an Arg54-->Cys substitution and acounterpart with an Asp277-->Ala substitution found in a compound heterozygote associated with severe hypophosphatasia. Biochem J. 2000 Jun 15;348 Pt 3(Pt 3):633-42. PMID: 10839996; PMCID: PMC1221107.

3. Del Angel G, Reynders J, Negron C, Steinbrecher T, Mornet E. Large-scale in vitro functional testing and novel variant scoring via protein modeling provide insights into alkaline phosphatase activity in hypophosphatasia. Hum Mutat. 2020 Jul;41(7):1250-1262. doi: 10.1002/humu.24010. Epub 2020 Mar 18. PMID: 32160374; PMCID: PMC7317754.

4. Fauvert D, Brun-Heath I, Lia-Baldini AS, Bellazi L, Taillandier A, Serre JL, de Mazancourt P, Mornet E. Mild forms of hypophosphatasia mostly result from dominant negative effect of severe alleles or from compound heterozygosity for severe and moderate alleles. BMC Med Genet. 2009 Jun 6;10:51. doi: 10.1186/1471-2350-10-51. PMID: 19500388; PMCID: PMC2702372.

5. Lia-Baldini AS, Muller F, Taillandier A, Gibrat JF, Mouchard M, Robin B, Simon-Bouy B, Serre JL, Aylsworth AS, Bieth E, Delanote S, Freisinger P, Hu JC, Krohn HP, Nunes ME, Mornet E. A molecular approach to dominance in hypophosphatasia. Hum Genet. 2001 Jul;109(1):99-108. doi: 10.1007/s004390100546. PMID: 11479741.

6. Michigami T, Tachikawa K, Yamazaki M, Kawai M, Kubota T, Ozono K. Hypophosphatasia in Japan: ALPL Mutation Analysis in 98 Unrelated Patients. Calcif Tissue Int. 2020 Mar;106(3):221-231. doi: 10.1007/s00223-019-00626-w. Epub 2019 Nov 9. PMID: 31707452.

7. Okawa R, Kokomoto K, Kitaoka T, Kubota T, Watanabe A, Taketani T, Michigami T, Ozono K, Nakano K. Japanese nationwide survey of hypophosphatasia reveals prominent differences in genetic and dental findings between odonto and non-odonto types. PLoS One. 2019 Oct 10;14(10):e0222931. doi: 10.1371/journal.pone.0222931. PMID: 31600233; PMCID: PMC6786601.
